# Utility of snout wipe samples for influenza A virus surveillance in exhibition swine populations

**DOI:** 10.1111/irv.12270

**Published:** 2014-07-10

**Authors:** Jody L Edwards, Sarah W Nelson, Jeffrey D Workman, Richard D Slemons, Christine M Szablewski, Jacqueline M Nolting, Andrew S Bowman

**Affiliations:** a Department of Veterinary Preventive Medicine, The Ohio State UniversityColumbus, OH, USA; b Ohio State University Extension, The Ohio State UniversityColumbus, OH, USA

**Keywords:** Diagnostic techniques and procedures, influenza A virus, surveillance, swine, zoonotic disease

## Abstract

**Background:**

Sporadic influenza A virus (IAV) outbreaks in humans and swine have resulted from commingling of large numbers of people and pigs at agricultural fairs in the United States. Current antemortem IAV surveillance strategies in swine require collecting nasal swabs, which entails restraining pigs with snares. Restraint is labor-intensive for samplers, stressful for pigs, and displeasing to onlookers because pigs often resist and vocalize.

**Objective:**

To evaluate the utility of snout wipes in exhibition swine as a method to make IAV surveillance efforts less intrusive, less labor-intensive, and more widely accepted among pig owners and exhibition officials.

**Methods:**

Three materials (rayon/polyester gauze, cotton gauze, and Swiffer® Sweeper dry cloths) were inoculated with IAV, and viral recoveries from these materials were quantified using qRT-PCR and TCID_50_ assays. In a field trial, paired cotton gauze snout wipes and gold standard polyester-tipped nasal swabs were collected from 553 pigs representing 29 agricultural fairs and the qualitative results of rRT-PCR and viral isolation were compared.

**Results and Conclusions:**

Viral recoveries from potential snout wipe materials ranged from 0·26 to 1·59 log_10_ TCID_50_/ml less than that of the positive control in which no substrate was included; rayon/polyester gauze performed significantly worse than the other materials. In the field, snout wipes and nasal swabs had high levels of agreement for both rRT-PCR detection and virus isolation. Although further investigation and refinement of the sampling method is needed, results indicate that snout wipes will facilitate convenient and undisruptive IAV surveillance in pigs at agricultural fairs.

## Introduction

Along with humans and birds, pigs are one of the major species to have played a role in the maintenance and emergence of influenza A viruses (IAVs) in the environment. Swine-origin IAVs also have been long considered a potential threat to animal and public health. The ongoing and rapid IAV evolution occurring in swine can result in novel IAV strain generation, which if zoonotic transmission and sustained person-to-person transmission occurs may result in pandemic disease. The longest sustained human-to-human transmission of a swine-origin IAV occurred in 2009. Influenza A(H1N1)pdm09 virus containing gene segment from North American and European swine lineages^1^ resulted in pandemic disease in both human and pigs and is still circulating among both populations. From 2007 to 2012, triple-reassortant viruses with genes of human, swine, and avian lineages were sporadically isolated from humans that did not always have direct contact with pigs.^2^–^6^ Recently, Xu *et al*.^7^ demonstrated that after several passages in pigs, an avian H7N9 virus increased in pathogenicity in pigs, showed increased human-type receptor binding, and demonstrated amino acid changes that increased similarity to human-isolated strains.

Agricultural fairs in the United States bring large numbers of people and swine together for extended periods of time and have been identified as a swine–human interface conducive to intra- and interspecies IAV transmission.^2^–^6^,^8^,^9^ From July through September 2012, 306 confirmed cases of influenza A (H3N2) variant virus (H3N2v) which contained the matrix gene from A(H1N1)pdm09 became the largest outbreak of human infections with a variant IAV since the 2009 H1N1 pandemic.^5^ Direct or indirect contact with swine was reported in 95% of these cases, with 93% of the cases reporting they had attended an agricultural fair within 4 days of illness onset.^5^,^10^ Molecular analyses of IAV isolates recovered from the pigs and people confirmed zoonotic transmission during the fair.^6^ These findings demonstrate a need for vigilant monitoring of IAV activity at the swine–human interface at agricultural fairs to develop and assess mitigation strategies for controlling IAV transmission in these settings.

Compounding the issue, subclinical IAV infections are common in exhibition swine. Between 2009 and 2011, pigs with clinical signs of influenza-like illness (ILI) were only observed at 16·7% of agricultural fairs where IAV was recovered from pigs.^9^ This finding highlights the importance of conducting active surveillance efforts which include healthy pigs along with those displaying signs of ILI. This type of exhaustive surveillance strategy necessitates more efficient and less disruptive sampling methods.

Current gold standard sampling procedures require the use of synthetic-tipped swabs to collect nasal mucosal secretions and surface epithelial cells.^11^ This procedure is labor-intensive, stressful to the pigs, and esthetically unpleasing to both swine owners and the public attending agricultural fairs because it necessitates the use of a restraining snare where a looped cable is placed in the mouth of the pig and tightened over its upper jaw.^12^ Because of these drawbacks, the majority of the IAV surveillance at agricultural fairs has occurred at or near the end of the exhibition period, after the animal competitions are completed. However, because we are unable to surveil IAV-infected pigs upon arrival or during the bulk of the exhibition period, little is known about IAV transmission occurring while fairs are in progress.

Non-woven fabric cleaning cloths have been successfully used as a method of collecting pooled samples in swine populations experimentally infected with IAV.^13^ In an attempt to overcome the limitations of colleting nasal swabs, we investigated the use of snout wipes as a non-invasive IAV surveillance method.

## Materials and methods

### *In vitro* wipe substrate comparison

To compare potential snout wipe substrates, 0·2 ml cell culture supernatant containing a total inoculum of 1·36 × 10^4^ TCID_50_ of A/swine/Ohio/12TOSU447/2012(H3N2) was inoculated in triplicate onto polyester-tipped swabs (catalog no. 23-400-111; Fisher Scientific, Waltham, MA, USA) and 2 in. × 2 in (5·08 cm × 5·08 cm) pieces of rayon–polyester blend gauze (catalog no. 893119; CVS Pharmacy Inc., Woonsocket, RI, USA); cotton gauze (catalog no. 441211; Covidien LLC, Mansfield, MA, USA); and Swiffer® Sweeper dry cloths (catalog no. 037000318224; Procter & Gamble, Cincinnati, OH, USA). The Swiffer® Sweeper dry cloths were cut to size using sterile technique. Following inoculation, the materials were placed into sterile high-density polyethylene (HDPE) storage pots (catalog no. 5005-0015; Thermo Scientific™ Nalgene™, Waltham, MA, USA) containing 5 ml viral transport media (VTM) consisting of brain heart infusion broth (BHIB) supplemented with 10 000 U/ml penicillin and 10 mg/ml streptomycin.^14^ Three vials with only VTM were used as negative controls. Three other vials containing only VTM (no sampling substrate) were directly inoculated with IAV as described above for positive controls (2·72 × 10^3^ TCID_50_/ml final viral concentration). All vials were frozen at ≤−70°C for at least 24 hours before testing was initiated. Vials were rapidly thawed in a 37°C dry bead bath. Viral recovery from each substrate was measured with qRT-PCR and TCID_50_ assay.

RNA was extracted from samples using the MagMAX™ -96 Viral RNA Isolation Kit (Life Technologies, Carlsbad, CA, USA) in conjunction with the MagMAX™ Express-96 Magnetic Particle Processor (Life Technologies) according to manufacturer's instructions with the exception of increasing starting sample volume to 100 μl and decreasing elution buffer to 50 μl per sample. qRT-PCR was performed using the VetMAX™-Gold SIV Detection Kit (Life Technologies) with the 7500 Fast Real-Time PCR System (Life Technologies) according to manufacturer's instructions. Quantitative standards were established using the manufacturer's supplied positive control.

Serial dilutions of VTM supernatant were inoculated in triplicate onto 96-well plates of serum-free adapted and maintained Madin-Darby canine kidney cells.^15^ TCID_50_ titers were calculated with the Reed & Muench method.^16^ The results were log_10_-transformed and compared using one-way anova, and Scheffe's *post hoc* test was used for multiple comparisons using stata/se 13.1 (StataCorp LP, College Station, TX, USA).

### Field trial

**Figure 1:**
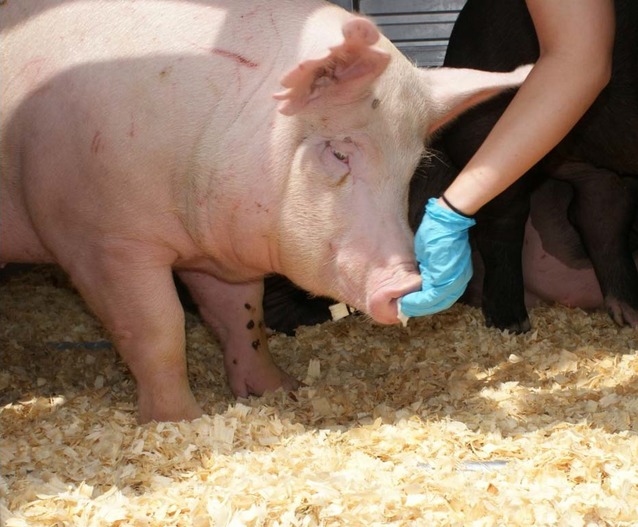
Collection of a snout wipe using gloved hand to wipe a 2 in. × 2 in (5·08 cm × 5·08 cm) sterile cotton gauze pad across the pig's snout. Restraint of the pigs was not needed. Emphasis was placed on contacting the gauze pad the external nares.

During June–August 2013, 553 paired nasal swabs and snout wipes were taken congruently from pigs at the end of 29 agricultural fairs across Ohio and Indiana. Each pig was restrained with a snare and a polyester-tipped swab was inserted into both nares of the pig after which the nasal swab was placed in an individual vial containing 1·8 ml of VTM. A snout wipe was collected from the same pig by wiping a 2 in. × 2 in (5·08 cm × 5·08 cm) sterile cotton gauze pad across the pig's snout with a gloved hand (Figure1). Gauze was placed in a sterile HDPE storage pot containing 5 ml VTM. Gloves were changed between pigs, and the snare was disinfected with Wexcide (Wexford Labs, Kirkwood, MO, USA). The order of nasal swab and snout wipe was alternated between pigs to correct for bias toward the sampling method used first on each pig. Nasal swab and snout wipe vials were frozen in the field on dry ice and stored at ≤−70°C until testing was conducted. Animals used in this study were included in protocol number 2009A0134-R1, which was approved by Institutional Animal Care and Use Committee of The Ohio State University.

The nasal swab samples were thawed in a 37°C dry bead bath, treated with amphotericin B (20 μg/ml), gentamicin sulfate (1000 μg/ml), and kanamycin sulfate (325 μg/ml) and vigorously agitated before centrifugation at 1200 ***g*** for 30 minutes at 4°C. Snout wipe samples were also thawed at 37°C after which they were treated with amphotericin B (22·5 μg/ml), gentamicin sulfate (1000 μg/ml), and kanamycin sulfate (325 μg/ml). A slightly higher concentration of amphotericin B was used for the snout wipes due to increased risk of fungal contamination from environmental debris. Due to the flat bottom of the HDPE vials and the volume of VTM, the snout wipes were not centrifuged like the nasal swabs.

Viral transport media supernatants from the nasal swab and snout wipes underwent testing by real-time reverse transcription PCR (rRT-PCR) using the VetMAX™-Gold SIV Detection Kit (Life Technologies) and were inoculated in quadruplicate onto monolayers of serum-free adapted and maintained Madin-Darby canine kidney cells, on 24-well plates.^15^ The rRT-PCR and virus isolation results from nasal swabs and snout wipes were cross-tabulated and compared using the kappa static.^17^

## Results

### In vitro

**Figure 2:**
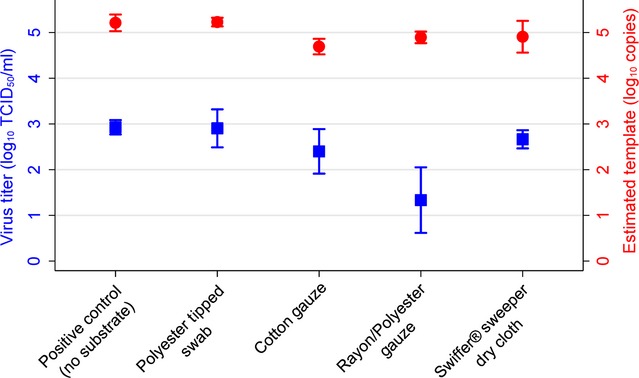
Comparisons of A/swine/Ohio/12TOSU447/2012(H3N2) recovery (log_10_ TCID50/ml) shown in blue and qRT-PCR detection (log_10_ copies) shown in red from each potential snout wipe and control substrate. The titers shown are the means from three replicates for each substrate, and error bars indicate the 95% confidence intervals.

When polyester swabs were compared with the positive controls, nearly identical qRT-PCR results were observed with 1·69 × 10^5^ and 1·63 × 10^5^ viral copies detected, respectively. All three potential wipe materials had significantly lower qRT-PCR results than the positive control (*P* = 0·01), but none of the three snout wipe substrates were statistically different from each other (Figure2) in terms of mean viral copy determined by qRT-PCR.

Viral recovery from the polyester-tipped swab inoculated with IAV (8·37 × 10^2^ TCID_50_/ml) was nearly identical to the positive control of IAV-spiked viral transport media (8·52 × 10^2^ TCID_50_/ml), confirming the gold standard use of nasal swabs for IAV isolation from pigs. Of the three potential snout wipe substrates tested, viral recovery from the rayon–polyester blend gauze was statistically lower (*P* = 0·001) than the other substrates with a 1·59 log_10_ decrease in TCID_50_/ml compared with the positive control (Figure2). In terms of viable virus recovery, the Swiffer® Sweeper dry cloths (4·66 × 10^2^ TCID_50_/ml) and the cotton gauze (2·68 × 10^2^ TCID_50_/ml) were not significantly different from each other; when compared to the positive control vials, these two substrates showed 0·26 and 0·52 log_10_ decreases in TCID_50_/ml, respectively.

### Field trial

Side-by-side analysis of 553 nasal swab and snout wipe samples showed substantial agreement for the detection of IAV by rRT-PCR (κ = 0·87). When the 553 samples were tested with rRT-PCR, 235 had positive results from both the nasal swab and snout wipe, 282 had negative results from both the nasal swab and snout wipe, and only 36 were not in full agreement with a negative result from one sampling method and a positive from the other (Table1). This produced an estimated sensitivity of 92·9% (95% CI: 88·9–95·7) for snout wipes when evaluated against nasal swabs.

**Table 1:** rRT-PCR detection influenza A virus from nasal swabs and snout wipes collected from swine at 29 county fairs

	Nasal swabs		
		
	Positive	Negative	Total
Snout wipes			
Positive	235	18	253
Negative	18	282	300
Total	253	300	553

Isolation of IAV in MDCK cells also showed strong agreement between the two sampling methods (κ = 0·82). Of the 553 samples, 151 were positive from both the nasal swab and snout wipe, 360 were negative from both the nasal swab and snout wipe, and only 42 were not in full agreement with a negative result from one sampling method and a positive from the other (Table2). The sensitivity of IAV isolation for snout wipes compared with the gold standard nasal swabs was 82·9% (95% CI: 76·7–88·1). The average Ct (threshold cycle) value for snout wipes that were RT-PCR and virus isolation positive was 24·32 (range: 16·83–34·77), whereas snout wipes that were RT-PCR positive and virus isolation negative had an average Ct value of 31·96 (range: 21·61–35·96).

**Table 2:** Isolation of influenza A virus from nasal swabs and snout wipes collected from swine at 29 county fairs

	Nasal swabs		
		
	Positive	Negative	Total
Snout wipes			
Positive	151	11	162
Negative	31	360	391
Total	182	371	553

## Discussion

While *in vitro* testing showed IAV recovery from cotton gauze snout wipes was lower than that from the gold standard polyester-tipped nasal swabs, field testing showed the use of snout wipes to be comparable to nasal swabs in terms of detection and isolation of IAV at the pig level. The decreased sensitivity in terms of IAV isolation must be considered, but we believe this cost is outweighed by the benefits of using snout wipes for IAV surveillance in pigs at agricultural fairs. The collection of snout wipes is likely not the ideal method for situations requiring high sensitivity for viable virus recovery (i.e., isolation of rare and valuable viral strains).

Collecting nasal swabs can be somewhat invasive and labor-intensive because it requires the use of a restraining snare. Pigs may try to resist or evade the snare and have the tendency to vocalize loudly while it is in use. The appearance of a struggling, squealing animal can be upsetting to the public and exhibitors attending the fair and may result in unfavorable views of surveillance efforts. Because pigs present at agricultural fairs are often enrolled in multiple competitions (showmanship, and/or market/breeding shows), a distressed pig is also likely to grieve owners for whom financial awards and future financial gain and notoriety from winning pigs are at stake. Because pigs are judged on overall presentation, owners may attribute lack of success in competition with the stress a pig endured during swab collection. Snout wipes do not require snaring and can be performed without upsetting the animals or owners and public in attendance. Additionally, the snout wipes do not need to be performed by a veterinary professional. A training video (http://go.osu.edu/snoutwipe) was prepared to allow exhibitors and family members to perform the sampling on their own pig and assist with IAV surveillance efforts. The decreased stress to the pigs and the ability to participate in sampling could increase willingness of owners to have their pigs sampled upon entry to the fair, an important step in preventing the transmission of IAV to other pigs and people at agricultural fairs.

Considering the viral recovery results of *in vitro* testing, Swiffer® Sweeper dry cloths were the top choice for use as a snout wipe material. Non-woven fabric cleaning cloths have been successfully used for collecting pooled samples in swine populations experimentally infected with IAV and are frequently used for a wide variety of environmental sampling procedures.^13^ However, the need to cut the cloths to a size suitable for individual animal testing and the necessity to achieve sterilization prior to utilization was laborious and costly. Additional challenges arose because they are comprised of proprietary materials, which complicate the ability to elucidate how the cloths might interact with the virus, hampering future investigations and finding equivalent replacements if they become unavailable. With viral recovery not statistically lower than Swiffer® Sweeper dry cloths, inexpensive cotton gauze is available in pre-sterilized individual packages, making it the most feasible choice of the three materials we compared. While synthetic fibers are thought to be the best swab material choice for viral sampling,^18^,^19^ our results show that in terms of viable viral recovery, the synthetic fiber gauze performed the worst of all the tested substrates. The reason for this surprising and contradictory finding is unknown, but indicates the need for broader investigation. The use of cotton as a viral collection substrate is mixed; cotton is generally believed to contain PCR inhibitors^20^ but on the other hand, cotton rope is the gold standard material for the collection oral fluids in swine herds.^21^ The data presented here show that while cotton had a decreased number of viral template copies detected with qRT-PCR as compared with the polyester-tipped swab (Figure2), cotton gauze agreed very well for the PCR detection of IAV in the field trial. The three materials investigated here are just a small subset of the extensive variety of fabrics (woven and non-woven) available today. Future, extensive, evaluation of other materials may allow for the identification of superior material better able to capture virus upon wiping and release it into the transport media.

The high agreement between nasal swabs and snout wipes demonstrated during our field trial could be attributed to time of sampling in relation to the course of infection. The discrepancies between PCR and virus isolation results are common in field surveillance efforts and can be attributed to the detection of non-viable virus or contamination of the samples with bacteria or other cytotoxic substances from the environment. Samples were taken at the end of the fair and are assumed to coincide with peak viral shedding in these environments. Sampling during times of peak viral shedding could potentially mask the decreased sensitivity of snout wipes due to the large amount of virus being shed by infected pigs because a qualitative outcome was measured in the field trial. The utility of snout wipes as a sampling method needs to be evaluated at other points of infection when viral shedding is lower. Decreased sensitivity will impact the determination of IAV prevalence during a fair.

Future studies evaluating snout wipe to transport media volume ratio are needed. Media volume affects virus and inhibitor concentration in the sample. The wipes also soak up the majority of the 5 ml of VTM, which lowers the volume available for testing and increases the concentration of inhibitors coming from environmental debris in the remaining unabsorbed sample. Because snout wipes provide a sample from a larger surface area, they often include more debris such as wood shavings, feed, and dirt which can be problematic for virus isolation. Media levels within the storage vial also affect whether the wipe stays immersed in media or absorbs much of the media volume and subsequently sticks to the bottom or side of the vial. A comparison of media volumes may be useful in identifying the best starting volume for the detection of sensitivity.

In addition to alternate media volumes, it may be useful to evaluate alternate sample storage vials and pots. The HDPE pots are much larger than standard cryovials and result in space limitations for freezer storage and handling/transporting in the field.

Future evaluation of the snout wipe method may also address its application with commercial swine. Recent diagnostic advances have pushed the detection of many pathogens in commercial swine settings toward collecting oral fluids, where animals penned together chew on a rope and deposit fluids which can then be harvested from the rope.^21^–^24^ This method does not work well at agricultural fairs and other settings where only one or two animals share a pen because they will not create an adequate volume of fluid to be collected. Also, virus isolation rates from oral fluids have been poor^22^ and can only give pooled results for a group of animals, not individuals. Our results show virus recovery from snout wipes to be promising and can also give results for individual animals. Even though rapid influenza diagnostic tests are notoriously poor for making individual animal diagnoses, future work should also investigate the use of snout wipes in commercially available rapid influenza diagnostic tests.

Because of the improved ease of use, decreased animal discomfort, and comparable levels of virus detection and isolation, the results of this pilot study support the use of snout wipes in place of nasal swabs for IAV surveillance in pigs at agricultural fairs. Due to the potential role swine play in the development of novel IAV strains and their proven ability to contract and transmit IAV strains bidirectionally to other pigs and humans at fairs, continuing aggressive surveillance is needed and warrants further investigation of this snout wipe method in pigs at agricultural fairs and in other settings.
